# Metopic triangle osteotomy in trigonocephaly Piezosurgery: a technical variant to control metopic emissary veins

**DOI:** 10.3171/2021.1.FOCVID20132

**Published:** 2021-04-01

**Authors:** Maria Licci, Pierre-Aurelien Beuriat, Alexandru Szathmari, Christian Paulus, Arnaud Gleizal, Carmine Mottolese, Federico Di Rocco

**Affiliations:** 1 Pediatric Neurosurgery,; 2 Centre de Référence Craniosténoses, and; 3 Maxillo-Facial Surgery, Hôpital Femme Mère Enfant, Université de Lyon, France

**Keywords:** metopic synostosis, osteotomy, emissary veins, metopic veins, complications

## Abstract

Premature fusion of the metopic suture results in trigonocephaly with variable degrees of anterior cranial fossa dysmorphia and craniofacial deformity. Different surgical corrective techniques that aim to reshape the forehead and enlarge the cranial volume have been described. Typical variations of the standard fronto-orbitary advancement carry the risk of relevant blood loss during frontal osteotomy, where paired emissary metopic veins are disrupted. The authors present a technical variant that preserves a bony triangle over the glabella to optimize control of these veins, which represent the major source of bleeding, and applies Piezosurgery to perform the osteotomies to minimize bone substance loss.

The video can be found here: https://vimeo.com/511536423.

## Transcript

The authors present a metopic triangle osteotomy in trigonocephaly surgery—a technical variant to control metopic emissary veins.

**0:29 Introductive Summary Slide**. Isolated, premature fusion of the metopic suture is increasingly common and second only to nonsyndromic sagittal suture synostosis in terms of frequency.^1,2^

Fronto-orbital remodeling remains the main treatment for metopic synostosis. Different patterns have been described.^3–6^

The perioperative morbidity of these surgical correction techniques is given by the risk of significant bleeding during osteotomies, due to the encasement of the superior sagittal sinus in the bone ridge and the presence of paired metopic transosseous emissary veins superior to the glabella that are associated with the fused metopic ridge.^7^

We present a technical variant of the frontal craniotomy that preserves a bony triangle over the glabella, preventing that the underlying veins are torn during elevation of the bony flap and therefore resulting in optimized hemostatic control.^7^

Piezosurgery is used to obtain precise cuts and minimize bone loss, which is particularly important at the level of the fronto-orbital bandeau to optimize aesthetic outcome.

**1:30 Informative Slide on Patient Selection**. For the purpose of extensive presentation of the surgical steps, the video demonstration is based on different cases of 7- to 9-month-old infants with trigonocephaly, all treated at our institution.

**1:42 Patient Positioning, Skull Exposure, and Outline of Osteotomies**. The patient is placed in supine position and the scalp is carefully elevated in a standard fashion through a bicoronal incision, paying particular attention when elevating the periosteal layer over the medial wall, the glabella, and the superior orbital ridge to reduce bleeding at this stage.

**1:58 Definition of the Metopic Triangle**. The design of the osteotomies preserves a bony metopic triangle over the classical fronto-orbital bandeau. This triangle, otherwise integrated in the frontal bone flap, lies 1 cm over the glabella with a 4-cm-wide base and a lateral extension of 2 cm to avoid lesioning of the metopic veins during craniotomy.

Frontal bone remodeling can be anticipated at this stage by outlining barrel-stave osteotomies as needed.

After planning of the osteotomies, a burr hole is placed on the midline at the upper limit, resulting in an overall height of about 4 cm.

**2:39 Frontal Osteotomy**. The dural plane is then dissected and the frontal osteotomy, following bone cuts previously outlined with surgical ink, can be performed either by Piezosurgery, as shown in this example, or by the use of a high-speed craniotome under cold water irrigation, or by a combination of both techniques, according to the surgeon's preference.

We usually use Piezosurgery when performing osteotomies of the forehead in order to minimize bone substance loss and to optimally preserve the fronto-orbital bandeau area. This surgical tool allows to obtain precise trajectories, creating sharp edges and clean, bone-sparing cuts.

**3:15 Frontal Bone Flap Removal**. The resulting frontal bone flap is mobilized with the aid of a surgical elevator, carefully dissecting the dura at the level of the anterior fontanelle, of the coronal sutures, and the upper part of the sagittal sinus. The area over the glabella, overlying the typically encased initial segment of sagittal sinus and the paired metopic veins, is still protected by the residual bony triangle at this point of the procedure.

**3:39 Forehead Exposition, Dura Mater Dissection, and Hemostasis**. The exposition that results from removal of the forehead bone flap now allows a meticulous dissection and detachment of the dura mater and the superior sagittal sinus from the inner part of the pathologically fused metopic ridge under direct control and a progressive coagulation of the frontal veins with optimized vision from both sides.

**4:00 Removal of the Metopic Triangle**. Next, the frontal osteotomy can be safely completed using Piezosurgery by removal of the residual osseous triangle, without relevant bleeding.

Final hemostasis over the superior sagittal sinus can be easily performed and metopic veins under the fronto-orbital bandeau can be safely cauterized.

**4:19 Fronto-Orbital Remodeling**. The surgical procedure then continues in a standard fashion, with the removal of the fronto-orbital bandeau for subsequent remodeling and advancement.

Different patterns of fronto-orbital remodeling, including forehead tilting, splitting of the frontal bones, and additional reshaping osteotomies, can be chosen, finally resulting in enlargement of the forehead.

With regard to the frontal bone, the authors’ technique that is shown typically consists in a fronto-orbital bandeau with temporal tongues and grooves, 180° tilting of the forehead, reshaping and placement of the metopic bony triangle at the level of the anterior fontanelle, followed by periosteal coverage and wound closure.

## Author Contributions

Primary surgeon: Di Rocco, Gleizal, Mottolese. Assistant surgeon: Licci, Szathmari, Paulus. Editing and drafting the video and abstract: Licci. Critically revising the work: Di Rocco, Beuriat. Reviewed submitted version of the work: Di Rocco, Licci, Beuriat. Supervision: Di Rocco.

## Supplemental Information

### Previous Presentations

The described operative technique has been previously published in Di Rocco et al.^7^
